# Air Will Find a Way: A Case Report and Literature Review on Tracheostomy-Induced Ectopic Air

**DOI:** 10.7759/cureus.42446

**Published:** 2023-07-25

**Authors:** Simrat K Batth, Gurkaranvir Singh

**Affiliations:** 1 Medicine, Elmhurst Hospital Centre (Icahn School of Medicine at Mount Sinai), New York, USA; 2 Medicine, Government Medical College Amritsar, Amritsar, IND

**Keywords:** mechanism of pneumoperitoneum, mechanism of pneumomediastinum, pneumomediastinum, pneumoperitoneum, tracheostomy complications

## Abstract

Tracheostomy can lead to various complications, one of which is ectopic air in different compartments of the body. Here, we present a rare case of tracheostomy-induced ectopic air: a combination of subcutaneous emphysema, pneumomediastinum, and pneumoperitoneum. This case also presents a literature review on some of the common mechanisms responsible for pneumomediastinum following tracheostomy and the mechanism of pneumoperitoneum following pneumomediastinum. Tracheal injury, tube-related complications, and alveolar rupture are common mechanisms that can lead to pneumomediastinum and subcutaneous emphysema after tracheostomy. Air can then dissect into the abdomen leading to pneumoperitoneum. Knowledge of the anatomic or embryologic development of the thoracoabdominal continuum can help understand the spread of air from one compartment to another. Investigation as simple as a chest X-ray, along with clinical features, can help identify these complications and be used to monitor the course.

## Introduction

Depending on where it is located, the air outside the aero-digestive tract is abnormal, and it is usually called emphysema, referring to trapped air/gas in the tissues or ectopic air/gas [1]. When air is present in the subcutaneous tissues, cervical, mediastinal, retroperitoneal, extraperitoneal, abdomen, and pelvis spaces, involving muscle fibers or interstitial tissues, it is abnormal, indicating a pathological process, and represents a challenge to search for the underlying etiology [1]. Abnormal gas/air beyond viscera and serosal spaces reaches its location following some anatomic boundaries, such as fascia, which helps search for the source [1]. This case report discusses ectopic air induced by tracheostomy and brings forth one of the rare combinations of complications: subcutaneous emphysema, pneumomediastinum, and pneumoperitoneum. Identifying these complications is crucial, as they can be life-threatening, whereas asymptomatic patients can be managed with conservative measures.

## Case presentation

**Figure 1:**
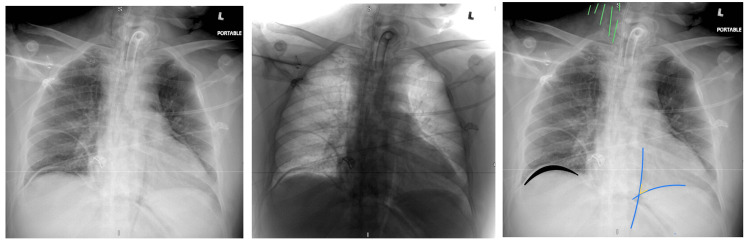
Portable chest X-ray done to confirm the position of the tracheostomy tube and rule out complications Tracheostomy and nasogastric tubes are in place. The lower right neck soft tissue appears heterogeneous with areas of ill-defined linear and nonlinear lucencies: subcutaneous emphysema (represented by the green marks). Air under the diaphragm on the right side can be seen: pneumoperitoneum (represented by a black marking). Multitudes of thin streaks can be seen in the mediastinum. Air can be seen along the left heart border (represented by red markings). Lucent band of gas that extends along the descending aorta and intersects the band that extends along the medial left hemidiaphragm, together forming a “V”: Naclerio’s V sign (represented by the yellow marking). Although this finding was originally described in association with esophageal rupture, it is not specific to this condition.

A 41-year-old male presented with dysarthria, aphasia, and right-sided hemiplegia due to left-sided pontine intracranial hemorrhage in the setting of a hypertensive emergency. He was managed with antihypertensive medications and intubated twice due to his inability to protect the airway and clear the secretions. He had an absent swallow reflex with excessive pooling of secretions, requiring frequent suctioning, leading to recurrent aspiration pneumonia. Finally, a decision for tracheostomy under bronchoscopic guidance was made. The patient was kept on mechanical ventilation while on sedation and remained hemodynamically stable. Chest X-ray was done to confirm the location of the tracheostomy tube, which showed unexpected findings of right neck subcutaneous emphysema, air in the mediastinum, and right subphrenic air (Figure 1).

**Figure 2:**
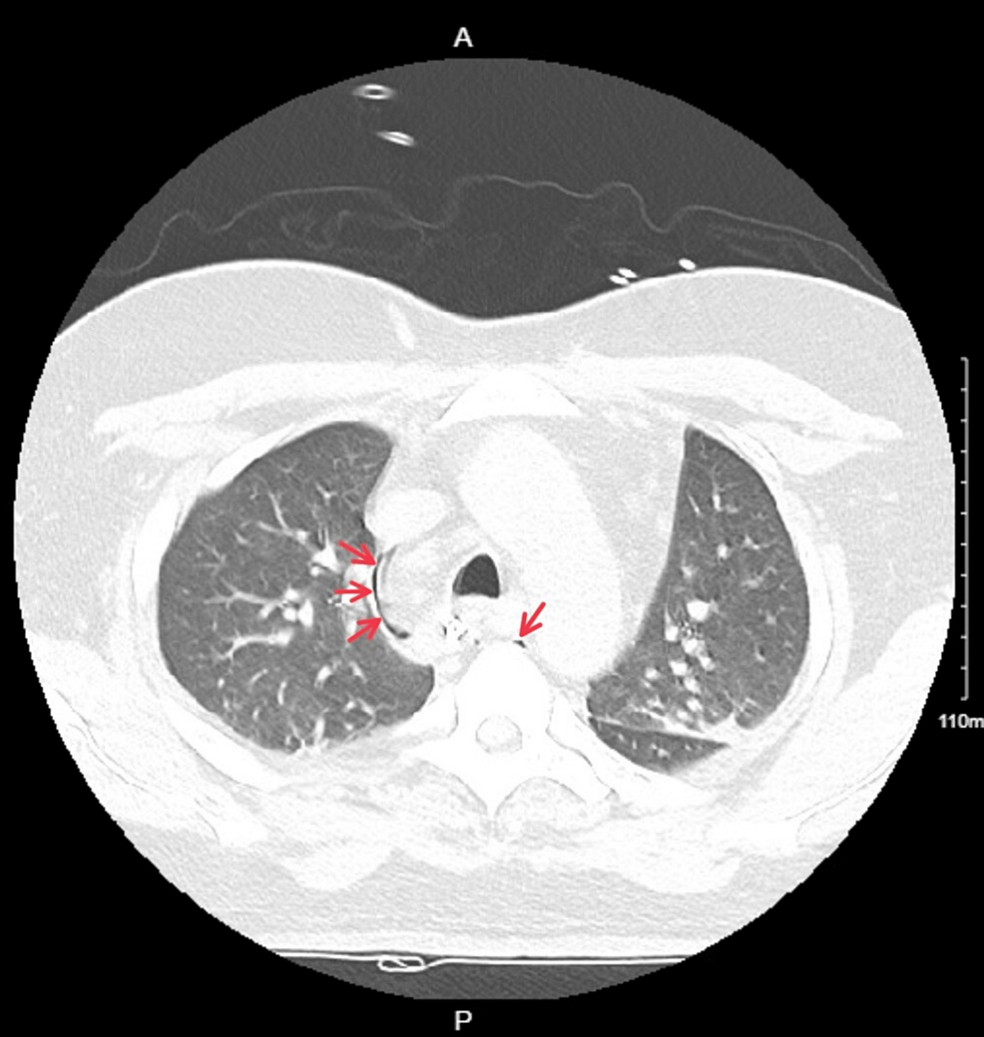
CT chest showing pneumomediastinum Air in the mediastinum can be seen as dark spaces (marked by red arrows).

**Figure 3:**
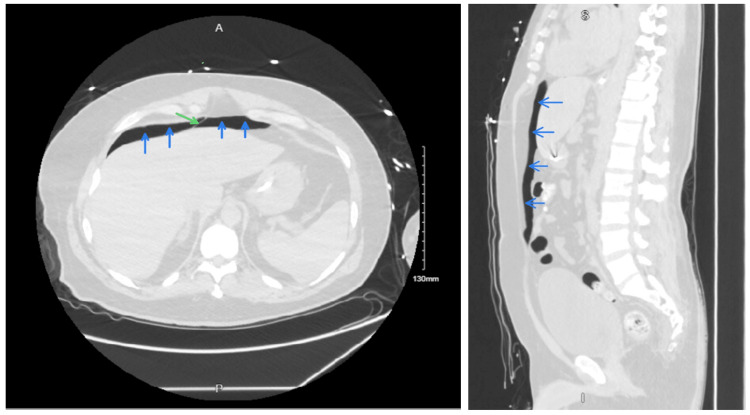
CT abdomen showing pneumoperitoneum Air in the peritoneum can be seen as a dark space (marked by blue arrows). The falciform ligament can be seen; the falciform ligament sign is marked in green. The CT abdomen is viewed in the lung window to make the pneumoperitoneum prominent.

CT chest and abdomen were done to confirm the findings and rule out any intra-abdominal pathology that could be responsible for or contributing to pneumoperitoneum (Figures 2, 3).

**Figure 4:**
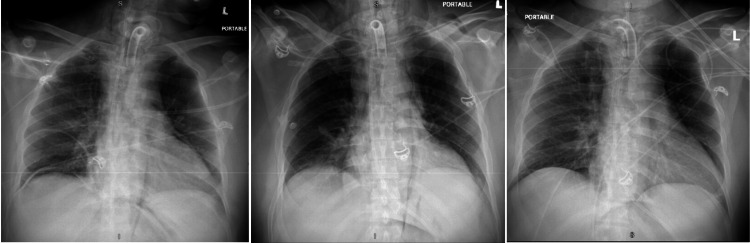
Resolution of subcutaneous emphysema, pneumomediastinum, and pneumoperitoneum seen on serial chest X-rays

CT chest and abdomen showed the presence of air in the mediastinum and peritoneum, and CT abdomen also showed an ulcer along the ventral aspect of the gastric antrum. The patient had stable vitals with insignificant physical examination findings except for mild abdominal distention on the abdominal exam. Due to sedation, other signs and symptoms of pneumomediastinum and pneumoperitoneum could not be found. Given his CT abdomen findings and the septic picture (febrile, elevated white counts from aspiration pneumonia), surgery was consulted due to concern for bowel perforation. However, it was unlikely that the pneumoperitoneum was from a bowel perforation. Diagnostic laparoscopy with esophagogastroduodenoscopy was undertaken to evaluate for perforated hollow viscus. Perforated hollow viscus was ruled out, confirming the diagnosis of pneumoperitoneum due to the dissection of air from the tracheostomy procedure. We were unable to identify the source of air in the mediastinum. Bronchoscopy done during tracheostomy and CT chest could not determine the source of pneumomediastinum. The patient remained hemodynamically stable throughout the course of his hospital stay and was managed with serial radiographic imaging showing improvement in pneumomediastinum and pneumoperitoneum (Figure 4).

## Discussion

This case brings forth one of the rare combinations of complications of tracheostomy: subcutaneous emphysema, pneumomediastinum, and pneumoperitoneum. There have been some case reports in the past on pneumomediastinum, pneumoperitoneum, and subcutaneous emphysema after tracheostomy [2-5]. The discussion of this case report focuses on the mechanism of pneumomediastinum due to tracheostomy and the development of pneumoperitoneum from pneumomediastinum.

Mechanism of pneumomediastinum after tracheostomy

Broadly, ectopic air after tracheostomy can be procedure-related (like the passage of air in the pretracheal fascia due to excessive dissection of tissue planes, tracheal wall injury/laceration), tracheostomy-tube related (blockage of the cannula, malpositioning, dislodgement, exchange of tracheostomy tubes) or alveoli-related (alveolar rupture from high ventilatory pressures, existing lung inflammation making it more susceptible to injury) [2-7]. The above-mentioned causes can lead to subcutaneous emphysema and pneumomediastinum with or without pneumothorax, which can track down to the peritoneum through hiatal openings in the diaphragm.

Posterior tracheal wall injury can commonly occur due to poor control of the guidewire and guiding catheter [8]. Most posterior wall tears are small and heal without further intervention, whereas large tears may present with airway bleeding, air leak around the tube, or into the mediastinum [8].

Tracheostomy tube malpositioning or dislodgement can lead to the creation of a false tract. Tracheostomy tube dislodgement, especially in the initial week, is a medical emergency because the absence of a mature tract may make the replacement of the tube difficult and create a false tract [8]. A number of causes may lead to tracheostomy tube dislodgement, like loose tracheostomy ties, accidental displacement while turning the patient, or self-extubation [8]. Tracheostomy tube obstruction may occur following the passage of the tube in a false lumen (paratracheal soft tissue), presenting with acute change in the respiratory status [8].

An alveolar rupture could be another source of air leaks. Some of the main causes of alveolar rupture are barotrauma from mechanical ventilation and inflammatory processes involving the alveoli, such as pneumonia which increases the susceptibility to rupture [7]. An increase in the intra-alveolar pressure and overinflation of the alveoli can lead to a large pressure gradient between the alveoli and interstitium [7,9]. This will result in a pressure gradient that can rupture the marginal alveoli, leading to air leakage into the interstitium, which can track along the perivascular and peribronchial sheath to the hilum of the lung and then to the low-pressure mediastinum [7,9]. Occasionally, the air can dissect the pericardium, retropharyngeal, retroperitoneal, and intraperitoneal space [9]. If the intra-mediastinal pressure rises abruptly or the decompression of the air dissecting into the subcutaneous air is insufficient to relieve the tension, the mediastinal parietal pleura may tear, resulting in pneumothorax [9].

Our patient was on mechanical ventilation after tracheostomy, which could have caused the alveolar rupture, with underlying lung inflammation from aspiration pneumonia predisposing to rupture. He also underwent two intubations in a week prior to tracheostomy, which might have created micro lacerations exacerbated by the tracheostomy procedure, leading to air leak, even though bronchoscopic examination of the tracheobronchial tree did not show any tracheal wall trauma. The tracheal damage may have occurred during intubations and extubations. We could not determine the source of the air leak in our patient but were able to rule out potentially life-threatening sources like bowel pathology, visible tracheal injury, and tracheostomy tube malpositioning.

Mechanism of pneumoperitoneum from pneumomediastinum

In order to understand the mechanism of air entry into the abdomen, it is important to know how the thoracic and abdominal cavities are actually a single cavity initially, and the development of the diaphragm separates these into two different cavities.

**Figure 5:**
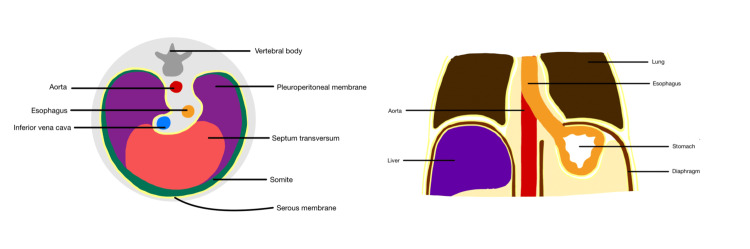
Continuation of the thoracoabdominal continuum (yellow line indicative of serous lining) The diaphragm is derived from three sources embryologically: the septum transversum, pleuroperitoneal folds, and somites. There are three main diaphragmatic apertures: esophageal, aortic, and inferior vena caval aperture. The continuity of the subserous space between the thorax and the abdomen is made possible by the aortic and esophageal hiatuses, two of the three main diaphragmatic openings [1,10,11]. The continuity of the subserous space is disrupted by the IVC wall's adhesion to the foramen's edges [1,10,11]. In addition to providing pathways for essential organs to travel from the thorax to the abdomen and vice versa, the subserous space also facilitates the spread of disease processes in both directions [1,10,11]. IVC: inferior vena cava Image credit: Simrat Kaur Batth

Based on embryologic development, an anatomic continuum exists between the spaces allowing air to spread along them [1]. During the early embryonic life, the coelomic cavity is formed, lined by a serous membrane, and deep into it lies the subserosal space; the integrity of this space is maintained during the division of the coelomic cavity, forming a thoracoabdominal continuum, which crosses the diaphragmatic hiatuses connecting the subperitoneal and subpleural portions of the subserous space [1]. There are three diaphragmatic hiatuses (Figure 5).

**Figure 6:**
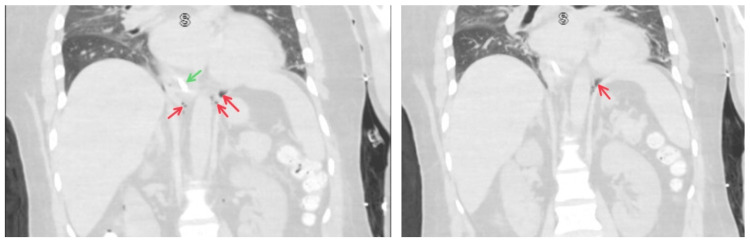
CT abdomen showing air dissecting down from thorax to abdomen Air (marked by red) can be seen along the descending aorta and esophagus (marked by green with a nasogastric tube in place).

The esophagus, vagus nerve, and esophageal vessels are all contained inside the esophageal hiatus, which is ventral and cranial to the aortic hiatus and allows for the continuity of the subserous space [1,10]. The subserous space, which contains the aorta, azygous vein, thoracic duct, and lymphatics, goes through the aortic hiatus, which is more dorsally and caudally located [1,10]. The subserous continuity found within the aortic and esophageal hiatuses represents a potential pathway for the transmission of disease (Figure 6) [10].

The vena caval foramen is the most ventral of the three and transmits only the inferior vena cava [1,10]. Because the wall of this vessel adheres to the edges of the foramen and disrupts the continuity of the subserous space, this opening does not present a risk for the spread of the disease [1,10]. The aortic hiatus maintain the subserous continuity between the thorax and abdomen, but further research of the surgical literature revealed that this continuity is also disrupted at the esophageal hiatus by the attachment of the phreno-esophageal ligament to the esophageal wall [11]. Small apertures can be found anteriorly between the sternum and the costal cartilage [10,11]. These apertures carry the superior epigastric branch of the internal mammary artery as well as lymphatics within the subserosal space at this location, offering a potential pathway for the disease to spread directly [10,11].

The radiographic findings are generated by the leaked air itself and an enhanced margin of mediastinal structures by the air [9]. Pneumopericardium, continuous diaphragm sign, continuous left hemidiaphragm sign, V sign at the confluence of brachiocephalic veins, ring-around-the-artery sign, thymic spinnaker-sail sign, extrapleural sign, and Naclerio's V sign are some radiographic signs of pneumomediastinum on chest X-ray [12]. Naclerio's V sign is a lucent band of gas that runs down the medial left hemidiaphragm and intersects a band that runs along the descending aorta to form a 'V' [12] (represented by the yellow marking in Figure 1). Although this result was initially associated with esophageal rupture, it is not unique to this condition [12]. Suphrenic air was appreciated in the portable chest X-ray for this patient, likely due to his semi-recumbent position as a precaution for aspiration pneumonia; otherwise, it's hard to see this sign on the supine chest X-Ray. CT scan is more sensitive than X-ray for detecting air.

Clinical features

Patients with pneumomediastinum and pneumoperitoneum can present with a variety of clinical features depending upon the acuity and degree of air leak, along with existing comorbidities of the patient. Patients who are under sedation, like our patient, may have subtle signs and symptoms or may not have any signs and symptoms of pneumomediastinum and pneumoperitoneum.

Patients who can communicate may complain of dyspnea, chest pain and may have tachycardia and tachypnea. There can be features of concomitant pneumothorax. Hamman’s sign, which consists of crackles or bubble sounds heard with each beat of the heart, can be heard on auscultation, especially if the patient is positioned in the left lateral position [9]. Hypotension can develop in tension pneumomediastinum due to decreased venous return and cardiac output, in which case, consultation with a cardiothoracic surgeon with surgical decompression may be needed [13]. In the case of barotrauma-induced pneumomediastinum, reducing the ventilatory pressures can hasten the resolution. Despite reports that a 100% oxygen supply promotes rapid absorption of emphysema - so-called “nitrogen washout,” the efficacy of such intervention is not conclusive, unlike in pneumothorax; therefore, routine use is not recommended for mediastinal emphysema [9].

In the case of pneumoperitoneum, patients may complain of abdominal discomfort and distention. Our patient had abdominal distention, but other features of pneumoperitoneum could not be determined since the patient was under sedation. Tension pneumoperitoneum can lead to tympany, tenderness, and abdominal compartment syndrome [5]. Operative intervention should not be undertaken in the absence of clinical and lab features of peritonitis. The appearance of pneumoperitoneum along with air at other places like the mediastinum, subcutaneous tissue, or pneumothorax after the tracheostomy procedure should be sufficient to delineate the etiology of pneumoperitoneum. Incidental findings on imaging, like suspicion of gastric ulcer on CT abdomen in our patient, can lead to unnecessary invasive procedures like laparoscopy and EGD.

Prevention

It is prudent to use a postoperative chest film to confirm appropriate tube placement [6]. Avoiding excessive tissue dissection of tissue planes at the time of tracheostomy, obstruction of the cannula, or assisted ventilation with excessive pressure resulting in the dissection of air along the pretracheal fascia are some other common preventative measures [6]. An adequate airway via bronchoscope or endotracheal tube decreases the negative inspiratory force, thereby diminishing the risk of air dissection along fascial planes [6]. Tight wound closure at the tracheostomy site can exacerbate the aforementioned conditions, which should be avoided [6]. Tube blockage is often avoided with postoperative care, including surveillance, humidification, and repeated gentle suctioning [6].

## Conclusions

The presence of air in different places of the body simultaneously could help make a linked diagnosis, reducing the number of investigations done to identify the source. Identification of ectopic air is important, as it can lead to life-threatening complications. But in patients who are asymptomatic, monitoring via serial physical exams and imaging as simple as chest X-rays can help save time and prevent unnecessary interventions. Basic knowledge of how thoracic and abdominal cavities are connected embryologically through different diaphragmatic hiatuses can help in understanding the possible paths that ectopic air can take. Meticulous tracheostomy procedures with knowledge of the anatomic continuum can prevent a majority of complications and quick management in case they occur.
